# Biofilm Dispersal in *Bacillus velezensis* FZB42 Is Regulated by the Second Messenger c-di-GMP

**DOI:** 10.3390/microorganisms13040896

**Published:** 2025-04-13

**Authors:** Meiyu Zhang, Shanyou Wu, Peng Chen, Lin Shao, Zizhu Shen, Yinjuan Zhao

**Affiliations:** 1 Collaborative Innovation Center of Sustainable Forestry in Southern China, Nanjing 210037, China; zhangmy16@njfu.edu.cn (M.Z.);; 2 College of Forestry, Nanjing Forestry University, Nanjing 210037, China

**Keywords:** c-di-GMP, biofilm, dispersal, *Bacillus velezensis* FZB42

## Abstract

Cyclic diguanosine monophosphate (c-di-GMP) is a second messenger that plays a crucial role in regulating biofilm development, yet the role in Gram-positive bacteria remains elusive. Here, we demonstrated that dispersed cells from biofilms of *Bacillus velezensis* FZB42 exhibit a unique phenotype and gene expression compared to planktonic cells. Transcriptomic analysis revealed 1327 downregulated and 1298 upregulated genes, among which the c-di-GMP phosphodiesterase coding *yuxH* gene was remarkably upregulated. Deletion of the *yuxH* gene led to elevated c-di-GMP levels accompanied by reduced amounts of “actively dispersed cells” from the pellicle and the capacity of motility. Deletion of *spoIIIJ*, *spo0J*, and *kinA* resulted in increased c-di-GMP levels and reduced biofilm dispersal ability. Also, the level of c-di-GMP was increased when adding the cues of inhibition biofilm dispersal such as glucose and calcium ions. Collectively, these present findings suggest the c-di-GMP level is negatively correlated with biofilm dispersal in *Bacillus velezensis* FZB42, which sheds new light on biofilm regulation in *Bacillus velezensis* FZB42.

## 1. Introduction

Biofilms are microbial communities producing substances in which bacteria cells are embedded within a self-produced extracellular polymeric matrix (EPS) composed of polysaccharides, proteins, and extracellular DNA [1,2]. The mechanism of biofilm formation is regulated by quorum sensing, signaling molecule networks, nutrient availability, hydrodynamic conditions, and intercellular communication [3,4]. Studying biofilms is important in various fields, including medicine, industry, and agriculture [5,6]. Biofilm development involves (1) the adhesion of cell-to-cell and cell-to-substrate, (2) the growth and division of cellular, (3) biofilm maturation with the formation of multiple layers and matrix production, and (4) detachment and dispersal of biofilm fragments or planktonic cells [7,8]. As the final stage of biofilm development, the dispersal stage involves bacteria detaching from an increasingly degraded environment and seeking a new habitat by degrading the biofilm matrix, synthesizing a large number of motility-associated proteins, and reverting to a planktonic lifestyle [9].

Like other microbial processes, biofilm dispersal is regulated by both environmental signals and intracellular signaling networks. Some environmental signals that trigger biofilm dispersal include metal ions, nutrients, and nitric oxide (NO) [7]. During this process, bacteria activate dispersal-associated signaling pathways, stop synthesizing biofilm matrix components, and instead synthesize motility-associated proteins such as produce extracellular factors (e.g., polysaccharide-degrading enzymes, proteases, nucleases, and biosurfactants) to degrade the biofilm matrix [7,10].

c-di-GMP broadly regulates biological processes such as bacterial growth, motility, adherence, biofilm formation, and pathogenicity, and its levels are coregulated by both endogenous factors [11] and exogenous factors [7,12]. Endogenous factors include internal bacterial regulatory mechanisms and molecular pathways that directly affect the synthesis and degradation of c-di-GMP. The synthesis of c-di-GMP is catalyzed by diguanylate cyclase (DGC) containing the GGDEF domain, which catalyzes GTP into c-di-GMP. Conversely, the degradation of c-di-GMP is mediated by phosphodiesterases (PDEs) possessing either the EAL or HD-GYP domains, which degrade c-di-GMP into pGpG or GMP [13,14]. Both the synthesis and degradation of c-di-GMP are further regulated by transcription factors. c-di-GMP signaling is usually achieved through interactions with specific c-di-GMP binding proteins (e.g., PilZ domain proteins) [13,15], which regulate cellular functions such as motility, adhesion, and biofilm formation by binding to c-di-GMP [16]. The feedback regulatory mechanisms for intracellular c-di-GMP levels ensure that the level matches the physiological needs of the cell, resulting in a complex self-regulatory system [17,18,19]. Environmental regulatory factors include the availability of carbon, nitrogen, and other nutrients [20]. Transcriptional regulatory c-di-GMP effectors modulate various bacterial physiological processes by directly regulating target gene expression in response to c-di-GMP [21]. For example, transcriptional regulatory c-di-GMP effectors, such as FleQ of *Pseudomonas aeruginosa* and MrkH of *Klebsiella pneumonia*, regulate the transcription of biofilm matrix and flagellum-related genes, thereby modulating biofilm formation and bacterial motility [22,23]. These factors work together through a complex signaling network to regulate bacterial physiological processes, including biofilm formation, motility regulation, drug resistance, and adaptation [12,24,25]. Numerous studies have demonstrated the key role of c-di-GMP in bacterial responses to environmental changes, particularly in pathogenicity, antibiotic resistance, and microbial community assembly. For example, in enteropathogenic *Escherichia coli* [26] or *Pseudomonas aeruginosa*, an increase in c-di-GMP levels was closely associated with biofilm formation, whereas decreased c-di-GMP levels promoted bacterial motility and pathogenicity [27,28]. By modulating c-di-GMP levels, bacteria can effectively form biofilms in different host environments and increase their survival in the host [12,24,29].

Changes in c-di-GMP levels are thought to represent a key regulatory mechanism [30]. Although many bacterial biofilms expand in response to environmental signals, studies of intracellular signaling pathways in reaction to external signals have focused on only a few bacterial species, such as *Pseudomonas aeruginosa* and *Staphylococcus aureus*, and most of these bacteria are Gram-negative [31,32,33,34,35,36,37,38]. Notably, it has been shown in previous studies that biofilms can help *B. velezensis* FZB42 play an important role in root colonization [39], as well as in growth promotion [40], stress tolerance, metabolite transport [41] and immune activity [42]. The present study revealed that in *B. velezensis* FZB42, increased intracellular c-di-GMP content inhibits biofilm dispersal. This study revealed a complex regulatory network of c-di-GMP during biofilm dispersal in *B. velezensis* FZB42, where c-di-GMP levels are not only influenced by intracellular genes but also modulated by external environmental factors, emphasizing the dynamic interactions between bacterial cells and their surroundings.

## 2. Materials and Methods

### 2.1. Bacterial Strains and Culture Media

*Bacillus velezensis* FZB42 was used to generate the Δ*yuxH*, Δ*spoIIIJ*, Δ*spo0J*, and Δ*kinA* mutants. The plasmids were constructed using *Escherichia coli* DH5α as the cloning host. The strains were stored in the −80 °C refrigerator in the laboratory. The Luria–Bertani (LB) medium solidified with 1.5% agar was supplemented with 100 µg/mL spectinomycin when necessary. The LBGM contained Tryptone 10 g/L, Yeast extract 5 g/L, NaCl 5 g/L, Glycerol 1% (*v*/*v*), MnSO_4_ 0.1 M, and additional agar 15 g/L was required for solid media. The plasmids used are shown in Table S1.

### 2.2. Construction of Knockout Strains

Isogenic mutants were generated via homologous recombination. The genomic DNA was extracted from wild-type FZB42 using Alkaline lysis as described previously [43] and served as the template for PCR. Upstream and downstream *yuxH*, *spoIIIJ*, *spo0J*, and *kinA* flanking regions were amplified with Prime STAR Max DNA Polymerase (R045Q, Takara, Kusatsu, Japan). Competent cells were prepared using the calcium chloride method as previously described [44]. The primers used are shown in Table S2. The plasmid pMarA (kindly provided by Prof. Fan) was used as a vector for transcription containing the spe^R^. The enzymes and reagents used are shown in Table S3. The plasmid construct was introduced into *B. velezensis* FZB42. Transformants were selected for double-crossover recombination on LB agar plates with 100 µg/mL spectinomycin. Response procedures and systems are shown in Supplementary Tables S4–S13.

### 2.3. Biofilm Dispersal of the Strains

Biofilm formation has been meticulously described in previously published studies [45]. *B. velezensis* FZB42 fresh single colonies were inoculated into the LB liquid medium. Then, they were incubated at 37 °C until the OD_600_ reached 1.0. Aliquots diluent (2 mL), the OD_600_ reached 2.0, was transferred into individual wells of a 24-well polystyrene plate and incubated at 25 °C for 36 h. The turbid liquid underlying the biofilm was aseptically aspirated using a sterile syringe. The optical density (OD_600_) of bacterial suspensions was measured by a spectrophotometer to estimate cell density.

### 2.4. Swarming Motility of B. velezensis FZB42

The cells of the *B. velezensis* FZB42 were cultured in 10 mL of LB liquid medium in the 100 mL conical flask until OD_600_ reached 1.0. A total of 1 μL of the bacterial culture was taken and inoculated onto the surface of the LB semi-solid medium (0.75% agar). The swarming agar plates were dried for 10 min in a laminar flow hood and incubated at 37 °C for 12 h. Then, they were swarming at room temperature to observe the bacterial motility.

### 2.5. Acquisition of Dispersed and Planktonic Cells

Dispersed cells were harvested using the method above for RNA extraction. Planktonic cells of *B. velezensis* FZB42 were harvested from the culture and incubated at 37 °C and 200 rpm in a shaker for 72 h when they reached the stationary phase.

### 2.6. Bacterial Transcriptome

Total RNA extraction of dispersed cells and planktonic cells was performed according to the Total RNA Extraction Kit (TR150-50, Tianmobio, Beijing, China). The collected samples were digested for DNA according to the instructions of Takara Recombinant DNase I (RNase-free). Removal of rRNA based on specific capture of ribosomal RNA sequences by streptavidin-coated magnetic beads (N512-01, Vazyme, Nanjing, China). The RNA was fragmented using VAHTS 2 × Frag/Prime Buffer (N402-01, Takara, Kusatsu, Japan). The synthetic double-stranded DNA was blunted at both ends and synthesized from RNA via reverse transcription. The 5′ ends were phosphorylated and ligated to adapters containing cohesive ends, and the 3′ ends with an A-overhang. The ligated product was amplified by PCR using specific primers. The PCR product was heat denatured to a single strand, which was then circularized with a bridging oligo to obtain a single-stranded circular DNA library. The single-stranded circular DNA libraries were then sequenced by the combinatorial probe anchor synthesis (cPAS).

### 2.7. Quantification of c-di-GMP Levels in Bacterial Biofilms by ELISA

As described previously [46], bacterial samples were collected and lysed with an ultrasonic homogenizer (JY92-IIN, Li-Cheng Ke Chuang, Ningbo, China) (the program was set to 200 w, ultrasound 3 s at 10 s intervals, repeated 30 times). The c-di-GMP concentration was tested according to the instructions of the cyclic di-guanosine monophosphate (c-di-GMP) ELISA Kit (Bllswbio, Shanghai, China).

### 2.8. Quantitative Real-Time PCR

The total RNA of the strains to be tested was extracted using the TRIzol method. The qPCR was performed according to the instructions of SYBR Green (FP209, TIANGEN, Beijing, China).

### 2.9. Stress Treatments

To assess the effects of ionic compounds on biofilm dispersal, mature biofilms were treated with sterile solutions (50 mM CuSO_4_, 2 mM FeSO_4_, 16 mM FeSO_4_, 2 mM FeCl_3_, 16 mM FeCl_3_, 1 mM Ca(NO_3_)_2_, 0.1 mM MnSO_4_, sterile deionized water), and incubated at 30 °C for 48 h. The OD_600_ was measured by a spectrophotometer.

Intracellular c-di-GMP levels in late-exponential-phase bacterial cultures (OD_600_ reached 0.6–0.8) grown in 10 mL LB cultures supplemented with glucose (1, 10, 100 mM) or calcium nitrate (1, 10, 100 mM) for 4 h were quantified using a c-di-GMP ELISA kit (Bllswbio, Shanghai, China) according to the manufacturer’s protocol.

### 2.10. Data Processing

The raw sequence data were preprocessed using SOAPnuke (v1.5.6) [47], which filtered out reads containing junctions (adapter contamination), reads with ambiguous base (N) content greater than 5%, and low-quality reads where >20% of bases had Phred scores < 15. The clean reads were obtained using Bowtie2 (v2.3.4.3) [48] to align the clean data to the reference genome. Differential gene detection was performed using DESeq2 (v1.4.5) [49] (or DEGseq [50] or PoissonDis [51]) provided that the *p*-value (FDR) < 0.05. Subsequent data analysis, mapping, and mining were performed using the Dr. Tom Multi-Organomics Data Mining System (https://biosys.bgi.com (accessed on 16 May 2024)) for data analysis, mapping, and mining. The transcriptome gene expression differences were obtained, and the fold-changes of biofilm-related genes of dispersed cells and planktonic cells were labeled into the pathway maps to obtain the pathway expression differences. Conducted singular value decomposition (SVD) used in R (v4.2.1). PCA plots via ggplot2 (v3.4.0) with ellipses representing 95% confidence intervals.

Data groups were analyzed using the one-way ANOVA and Student’s *t*-test to evaluate associations between independent variables, and the *p* values were calculated. Three independent trials were conducted in triplicates for each experiment, where the results were shown as the mean  ±  standard deviation. The bar chart and line graph were expressed through GraphPad Prism 8.0 obtained.

## 3. Results

### 3.1. High-Level Expression of c-di-GMP-Degrading Enzymes in Dispersed Biofilm Cells

**Figure 1:**
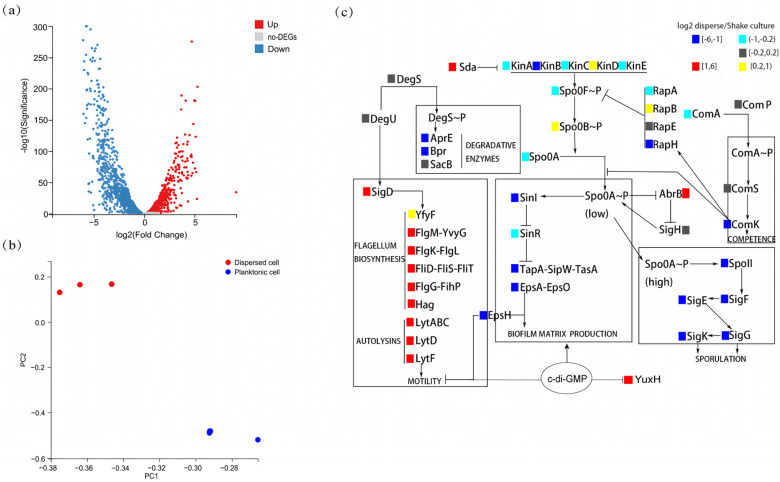
Transcriptomic profiling of dispersed versus planktonic cells. (**a**) Volcano plot of differentially expressed genes (DEGs). Red: upregulated in dispersed cells (log_2_(fold-change) > 1, FDR-adjusted *p*-value < 0.05); blue: downregulated in dispersed cells (log_2_(fold-change) > 1, FDR-adjusted *p*-value < 0.05); gray: non-significant. (**b**) Principal component analysis (PCA) of dispersed cells versus planktonic cells of transcriptomes (n = 3 biological replicates per group). (**c**) Heatmap of biofilm-associated DEGs. Color key: red: upregulated in dispersed cells (log_2_(fold-change) > 1, FDR-adjusted *p*-value < 0.05). Yellow: upregulated in dispersed cells (0.2 < log_2_(fold-change) ≤ 1, FDR-adjusted *p*-value < 0.05). Blue: downregulated in dispersed cells (log_2_(fold-change) < −1, FDR-adjusted *p*-value < 0.05). Cyan: downregulated in dispersed cells (−1 ≤ log_2_(fold-change) < −0.2, FDR-adjusted *p*-value < 0.05). Gray: non-significant (FDR-adjusted *p*-value ≥ 0.05).

To explore the differences between the characteristics of biofilm-dispersed and planktonic *B. velezensis* FZB42 cells, we analyzed the differences between them at the gene expression level. The results of the transcriptome analysis revealed differences in gene expression between biofilm-dispersed cells and planktonic cells (Figure 1a). The transcript levels of 1298 genes were significantly upregulated, and those of 1327 genes were significantly downregulated. Principal component analysis (PCA) revealed that the three dots representing biological replicates of biofilm-dispersed cells are clustered in the upper part, and the three dots representing planktonic cells are clustered in the lower part. And the inter-group variation was greater than the within-group differences (Figure 1b). Specifically, differential expression analysis of biofilm-associated genes shows that biofilm-dispersed cells presented increased expression levels of motility-related genes (e.g., *lytD* and *hag*) and chemotaxis-related genes (e.g., *pilA* and *fliC*), whereas planktonic cells presented increased expression levels of genes related to the synthesis of matrix biosynthesis (e.g., *epsA* and *tapA*) and genes related to spore formation (e.g., *spoIIA* and *sigK*). Overall, biofilm-dispersed cells presented relatively high expression levels of antibiotic-related genes (e.g., *sfp* and *yczE*), but the expression of some genes (e.g., *srfAA* and *bmyC*) was reduced (Figure 1c). At the same time, we also found a significant increase in the expression of YuxH, which has a c-di-GMP EAL-domain phosphodiesterase, suggesting that biofilm dispersal in *B. velezensis* FZB42 may be correlated with c-di-GMP.

### 3.2. c-di-GMP Regulates Multicellular Behaviors During Biofilm Dispersal

**Figure 2:**
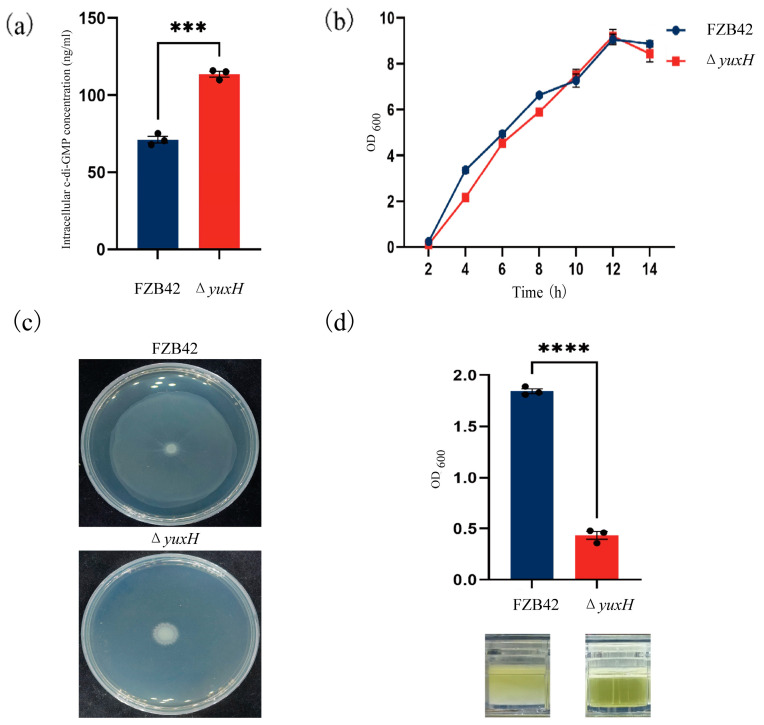
Phenotypic characterization of *yuxH* knockout versus wild-type *B. velezensis* FZB42. (**a**) Intracellular c-di-GMP levels in the *yuxH* knockout strain compared to the wild-type FZB42 (*** presents *p* < 0.001). (**b**) Comparison of the growth curves of the *yuxH* knockout strain and the wild-type FZB42. (**c**) Swarming motility assay on 0.75% agar LB plates of the *yuxH* knockout strain and the wild-type FZB42. (**d**) OD values of the turbidity liquid underlying the biofilm of the wild-type FZB42 and *yuxH* knockout strains and corresponding graphs of the turbidity liquid underlying the biofilm in 24-well plates after 36 h of standing and the state of the turbidity liquid underlying the biofilm. Error bars represent the SD of data from three independent experiments. **** presents *p* < 0.0001.

To investigate how the *yuxH* gene affects c-di-GMP homeostasis, we constructed a *yuxH* deletion mutant in *B. velezensis* FZB42 and compared intracellular c-di-GMP levels between the Δ*yuxH* and wild-type FZB42 strains. The results revealed that the Δ*yuxH* strain intracellular c-di-GMP concentration was 113.6 ng/mL, whereas the wild-type FZB42 was only 71.2 ng/mL, which demonstrated that *yuxH* encodes a functional c-di-GMP phosphodiesterase (PDE) essential for modulating c-di-GMP homeostasis in this bacterium (Figure 2a). To assess the growth phenotype, we determined the 14 h growth curves analysis of the knockdown strain and the wild-type strain. The results revealed that the effect of Δ*yuxH* on bacterial growth was not significant (Figure 2b). We also compared the growth status of the Δ*yuxH* strain with that of the wild-type strain on semi-solid plates. The results showed that the wild-type FZB42 strain retains full motility capacity; however, Δ*yuxH* significantly inhibited bacterial motility (Figure 2c). Furthermore, after 36 h of continuous incubation at 37 °C in LBGM, the turbidity liquid was underlying the biofilm of *B. velezensis* FZB42, whereas the bacteria culture of the knockout strain remained clear. By measuring the OD value at a wavelength of 600 nm, the absorbance value of the turbid liquid underlying the *B. velezensis* FZB42 culture was significantly greater than that of the Δ*yuxH* (Figure 2d). These findings indicated that Δ*yuxH* mutants with elevated c-di-GMP levels display impaired motility and biofilm dispersal; however, it had no significant effect on bacterial growth.

### 3.3. External Signals Can Inhibit B. velezensis FZB42 Biofilm Dispersal by Increasing Intracellular c-di-GMP Levels

**Figure 3:**
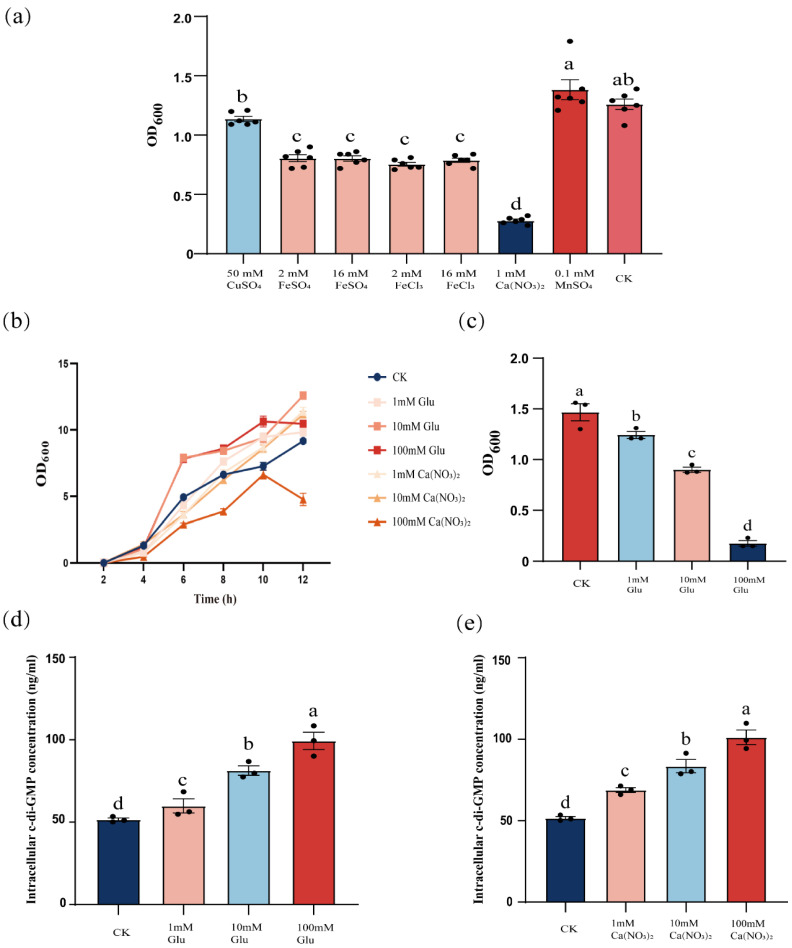
Effect of glucose and calcium ions on the dispersal of *B. velezensis* FZB42 biofilm. (**a**) Effect of different ions on biofilm dispersal of *B. velezensis* FZB42. (**b**) Growth curves of *B. velezensis* FZB42 in LB medium with different concentrations of glucose and calcium ions. (**c**) Effect of different concentrations of glucose on the dispersal of *B. velezensis* FZB42 biofilm. (**d**) Effect of different concentrations of glucose on c-di-GMP. (**e**) Effect of different concentrations of calcium ions on c-di-GMP. Error bars represent the SD of data from three independent experiments. Different lowercase letters above bars indicate statistically significant differences (one-way ANOVA with Tukey’s HSD post hoc, *p* < 0.05).

Building on the established role of *yuxH* in c-di-GMP homeostasis, we hypothesized that extracellular cues may synergistically regulate c-di-GMP dynamics. To test this hypothesis, wild-type cells were exposed to different external signals and quantified changes in intracellular c-di-GMP levels. We observed that intracellular c-di-GMP levels increased significantly when cells were exposed to specific external signals, such as certain environmental stimuli or chemicals. To verify the effects of different external signals on biofilm dispersal, we tested the effects of ions such as copper sulfate, ferrous sulfate, ferric chloride, calcium nitrate, and manganese sulfate on biofilm dispersal and examined the effects of different concentrations of glucose on biofilm dispersal. The results showed that the addition of iron and calcium ions decreased the OD_600_ value, and compared with the control group, there was a significant difference. The addition of manganese and copper ions did not have a significant difference, and calcium ions had the most significant inhibitory effect on biofilm dispersal (Figure 3a). Not only were calcium ions able to reduce the OD_600_ of *B. velezensis* FZB42, but glucose also had the reduction (Figure 3c). And to exclude that the two were OD_600_ effects due to the inhibition of the growth of *B. velezensis* FZB42, we determined the growth curves, and except 100 mM calcium nitrate, 1–100 mM glucose and 1–10 mM calcium nitrate affected the *B. velezensis* FZB42 growth without negative effects (Figure 3b). In the above results, we know that the level of c-di-GMP could affect the dispersal of *B. velezensis* FZB42, and the addition of glucose and calcium ions also had an effect on dispersal, so we explored whether the two mediate the changes in the level of c-di-GMP. We found that the intracellular c-di-GMP level of *B. velezensis* FZB42 increased significantly with increasing concentrations of exogenously added calcium nitrate and glucose (Figure 3d,e), suggesting that the inhibition of *B. velezensis* FZB42 biofilm dispersal by glucose, together with calcium nitrate, was also regulated by influencing the intracellular c-di-GMP concentration. Demonstrating that these stimuli inhibit biofilm dispersal by elevating c-di-GMP levels, paralleling the Δ*yuxH* mutant phenotype.

### 3.4. Biofilm-Associated Proteins Mediate c-di-GMP-Regulated Biofilm Dispersal Processes

**Figure 4:**
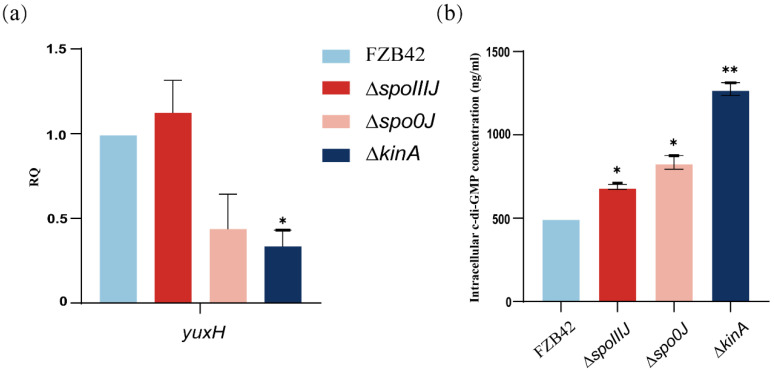
Expression of c-di-GMP synthesis degradation-related genes and intracellular c-di-GMP levels of *B. velezensis* FZB42, ∆*spoIIIJ*, ∆*spo0J*, and ∆*kinA*. (**a**) c-di-GMP expression of genes related to synthetic degradation. RNA was extracted from a 36 h old culture, and the expression of *yuxH* genes was quantified by qPCR. Fold-change in expression was normalized according to the expression of the reference gene *gyrA* in *B. velezensis* FZB42. Results represent the mean SD of three measurements performed on three independent RNA extractions. * presents *p* < 0.05 (one-way ANOVA with Dunnett’s test). (**b**) Intracellular c-di-GMP levels of FZB42, ∆*yuxH*, ∆*spoIIIJ*, ∆*spo0J* and ∆*kinA*. Results represent the mean SD of three measurements performed on three independent RNA extractions. * presents *p* < 0.05, ** presents *p* < 0.01 (one-way ANOVA with Dunnett’s test).

To investigate whether the *spoIIIJ*, *spo0J*, and *kinA* deletions affect the intracellular c-di-GMP level, we analyzed the changes in the expression of the *yuxH* gene in different mutant strains. The expression of *yuxH* was significantly lower in the Δ*kinA* compared with wild-type FZB42, suggesting that the predicted expression of *yuxH* is lower in these mutant strains (Figure 4a). Therefore, we hypothesized that c-di-GMP levels might be elevated in the Δ*spoIIIJ*, Δ*spo0J*, and Δ*kinA* strains, particularly in the Δ*kinA* strain. The analysis revealed that intracellular c-di-GMP levels were significantly higher in Δ*spoIIIJ*, Δ*spo0J*, and Δ*kinA* strains than in the wild-type FZB42 strain (Figure 4b). These results confirm that *yuxH* is indeed associated with c-di-GMP degradation. Specifically, the c-di-GMP levels of Δ*spoIIIJ* and Δ*spo0J* were comparable to those of the Δ*yuxH*, whereas the c-di-GMP levels of the Δ*kinA* were significantly higher than those of the Δ*yuxH*, suggesting that *kinA* not only inhibits c-di-GMP degradation by reducing the expression of *yuxH* but also affects c-di-GMP synthesis by regulating c-di-GMP levels. Consistent with the qPCR analysis results, these results indicate that deletion of the *spoIIIJ*, *spo0J*, and *kinA* genes significantly increased intracellular c-di-GMP levels.

## 4. Discussion

*Bacillus velezensis* FZB42 is a beneficial Gram-positive bacterium closely related to *Bacillus subtilis* in genetic taxonomy [52]. As a type strain of plant growth-promoting rhizobacteria (PGPR), it has substantial application potential for industrial production and agricultural protection. The strain can produce more than 10 antimicrobial compounds, inhibit a broad spectrum of plant pathogens, and assemble resilient biofilms [53]. c-di-GMP is a crucial signaling molecule that maintains biofilm stability and dynamic balance through its biosynthetic and catabolic enzymes. It is often referred to as a conversion molecule that regulates the lifestyle transition between motility and biofilm formation [54]. Specifically, c-di-GMP levels are linked to the switch between planktonic and biofilm states while also influencing cell cycle, morphology, and pathogenicity in various species [20,24,25,55].

Transcriptional profiling revealed distinct patterns between biofilm-dispersed and planktonic cells, consistent with previous reports by Krober [41]. We focus on the reduced aggregation capacity of dispersed cells rather than planktonic cells. Although this phenomenon can be thought to be caused by the reduced surfactin production, we found that the upstream genes related to the regulation of biofilm (e.g., *degS*, *spo0F*) were not significantly changed compared to the downstream genes (e.g., *epsA*, *tapA*). This is not in line with the signaling process of the biofilm formation stage, and we hypothesize that the biofilm signal transduction pathways may behave differently than during biofilm formation. The notable upregulation of YuxH prompted further investigation, suggesting that biofilm dispersal in *B. velezensis* FZB42 may be associated with c-di-GMP levels [29,56]. In Gram-negative bacteria, enzymatic degradation of biofilm matrix components induces dispersal [53]. The active degradation of c-di-GMP by phosphodiesterases (PDEs) can induce the production of matrix-degrading enzymes that facilitate biofilm detachment [20,54,55]. These enzymes regulate biofilm formation and spreading by hydrolyzing intracellular c-di-GMP molecules, thereby reducing their level. Our experimental data corroborate transcriptomic analyses. c-di-GMP degradation is a key mechanism that facilitates the transition of bacteria from the biofilm state to the planktonic state [25]. In the results comparing the Δ*yuxH* mutant with wild-type FZB42, we found that the Δ*yuxH* mutant causes an increase in c-di-GMP levels accompanied by a reduction in biofilm dispersal capacity, which is consistent with the hypothesis that we carried out earlier at the transcriptome level. Low c-di-GMP levels increase cell motility and facilitate biofilm dispersal. A correlation between high c-di-GMP levels and biofilm formation, or between low c-di-GMP levels and motility, has been demonstrated in several bacterial species [57]. The role of c-di-GMP in biofilm development has been highlighted in multiple studies, including the Gac/Rsm cascade, which controls biofilm formation through c-di-GMP signaling, the SagS pathway, which regulates biofilm antimicrobial resistance via c-di-GMP signaling, and the Las-mediated quorum sensing system, which controls biofilm formation and collective movement through c-di-GMP signaling [58,59,60,61]. For example, *Pseudomonas syringae* Δ*pscA* exhibits present c-di-GMP levels, leading to altered biofilm development in plants [62]. c-di-GMP-regulated biofilm determinants include extracellular polysaccharide production, surface adhesin expression, antimicrobial resistance, stress responses, and secondary metabolite production [25]. In *Pseudomonas aeruginosa* PA68, the Δ*dipA* mutant shows elevated intracellular c-di-GMP levels, whereas disruption of this gene in the PA68 strain results in reduced motility and suppressed diffusion behavior [31,63]. In *Pseudomonas malodorata*, cleavage of the surface-adherent LapA protein in a c-di-GMP-dependent manner mediates biofilm diffusion [64,65]. Notably, enzymes such as phosphodiesterases and proteins from the GGDEF family are expressed at significantly higher levels in dissociated cells, underscoring the role of c-di-GMP degradation in biofilm dispersal [66]. These differences, which may depend on the specific experimental conditions, suggest that the c-di-GMP signaling pathway is regulated by various mechanisms. Our findings align with those of Liu [67] and Katharios-Lanwermeyer [68], who also proposed that c-di-GMP plays a central role in regulating biofilm dispersal.

c-di-GMP precision controls bacterial community behavior by allosterically modulating the activity of biofilm-associated proteins, maintaining a delicate balance between biofilm formation and dispersal. Unlike intracellular bacteria, which reside in stable ecological niches, most free-living bacteria face complex and rapidly changing environments. External signals play crucial roles in regulating bacterial biofilm dynamics, potentially via the c-di-GMP signaling pathway. Biofilm dispersal is regulated by many external signals, such as temperature, pH, nutrients, oxygen, and ions [69,70]. It is supported by our findings that extracellular factors such as glucose and calcium ions significantly inhibit biofilm dispersal in *B. velezensis* FZB42, with concomitant increases in intracellular c-di-GMP levels. Calcium ions have been shown to activate the GGDEF/EAL domain [71]. In *Bacillus*, calcium ions are sequestered during spore formation by binding to Ca-DPA, and bacteria take up calcium ions from the environment as part of their spore formation process [72]. Interestingly, Nishikawa [73] reported that calcium ions regulate *B. subtilis* biofilm dispersal, although they do not significantly affect the expression of biofilm matrix-associated genes (e.g., *epsA*, *tapA*). Our study further supports the concept that bacteria dynamically regulate c-di-GMP levels in response to environmental cues, enabling them to adapt to various growth and survival conditions [7,25]. Compared with the other mutants, the deletion mutants *spoIIIJ*, *spo0J*, and *kinA* presented significant changes in intracellular c-di-GMP levels. The *kinA* mutant strain presented notably increased c-di-GMP levels, and the analysis indicated that *kinA* may inhibit biofilm dispersal by regulating c-di-GMP synthase activity, thereby affecting the overall c-di-GMP levels. These findings suggest that bacteria can adjust biofilm stability in response to environmental changes by regulating the expression of these genes [12,24].

Although we demonstrated that the phenomena affecting biofilm dispersal in *B. velezensis* FZB42 are related to c-di-GMP level-related aspects, the key mechanistic aspects of c-di-GMP signaling remain unresolved. While the current findings provide preliminary mechanistic insights into c-di-GMP-mediated biofilm dispersal in *B. velezensis* FZB42, robust validation through expanded experimental replicates under diverse physiological conditions will be essential to consolidate these observations.

## 5. Conclusions

**Figure 5:**
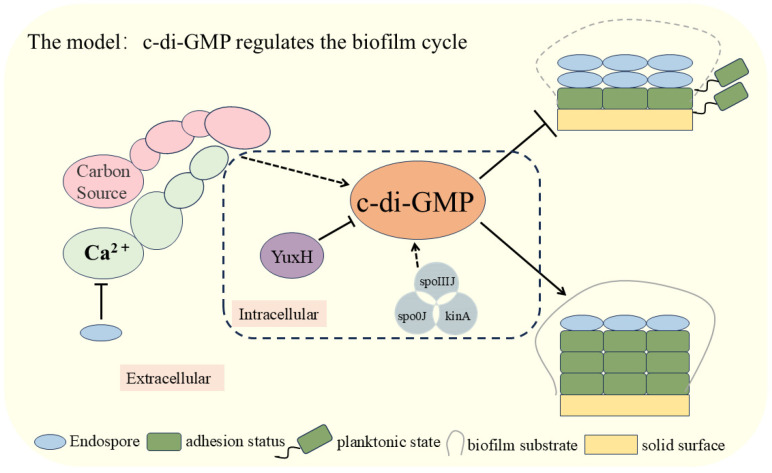
Modeling the involvement of c-di-GMP in the regulation of the biofilm cycle of *Bacillus velezensis* FZB42.

In this study, we elucidated the critical role of c-di-GMP in the biofilm dispersal process of *B. velezensis* FZB42 (Figure 5). We analyzed the effect of knocked-out genes that influence the level of c-di-GMP interrelationships among external cues. The experimental results demonstrated that external signals, such as glucose and calcium ions, significantly inhibited the biofilm dispersal of *B. velezensis* FZB42 by increasing intracellular c-di-GMP levels. Specifically, the pivotal role of the c-di-GMP-degrading enzyme YuxH in biofilm stability and dissociation was confirmed. Furthermore, deletion of the *spoIIIJ*, *spo0J*, and *kinA* genes, which correlate with biofilm development, significantly elevated c-di-GMP levels. The findings of this study enhance our understanding of the c-di-GMP regulatory mechanism and provide new directions for investigating bacterial biofilm dispersal. Future research could explore the relationship between additional exogenous signals and c-di-GMP and how these signals can be leveraged to regulate bacterial biofilm behaviors.

## Data Availability

The original contributions presented in this study are included in the article/Supplementary Materials. Further inquiries can be directed to the corresponding author.

## Supplementary Materials

The following supporting information can be downloaded at: https://www.mdpi.com/article/10.3390/microorganisms13040896/s1. Table S1: The plasmids; Table S2: The Primers required for amplification and validation of Δ*yuxH*, Δ*spoIIIJ*, Δ*spo0J*, Δ*kinA*; Table S3: The reagents and enzymes; Table S4: Preparation of PCR reaction solution of Δ*yuxH*; Table S5: PCR reaction Conditions of Δ*yuxH*; Table S6: A-tailed reaction system for PCR products; Table S7: PCR product and linearized T-Vector pMD-19 ligation reaction system; Table S8: Amplified linear fragment PCR reaction system; Table S9: Amplified linear fragment PCR reaction conditions; Table S10: Overlap extension PCR reaction system of Δ*spoIIIJ*, Δ*spo0J*, Δ*kinA*; Table S11: Overlap extension PCR reaction conditions Δ*spoIIIJ*, Δ*spo0J*, Δ*kinA*; Table S12: Verification PCR reaction system Δ*spoIIIJ*, Δ*spo0J*, Δ*kinA*; Table S13: Verification PCR reaction conditions Δ*spoIIIJ*, Δ*spo0J*, Δ*kinA*.
