# Supplementary material for: Biofilm Dispersal in Bacillus velezensis FZB42 Is Regulated by the Second Messenger c-di-GMP
Source: Microorganisms. 2025 Apr 13;13(4):896. doi: 10.3390/microorganisms13040896 (PMC12029170; doi:10.3390/microorganisms13040896)
Supplement: Supplementary file 1 [file microorganisms-13-00896-s001.zip › microorganisms-3505559-supplementary.pdf]

Supplementary Materials

Table S1 The plasmids.

Table S2 The Primers required for amplification and validation of Δ*yuxH*、Δ*spoIIIJ*、Δ*spo0J*、Δ*kinA*.

Table S3 The reagents and enzymes.

Table S4 Preparation of PCR reaction solution of Δ*yuxH*.

Table S5 PCR reaction Conditions of Δ*yuxH*.

Table S6 A-tailed reaction system for PCR products

Table S7 PCR product and linearized T-Vector pMD-19 ligation reaction system

Table S8 Amplified linear fragment PCR reaction system.

Table S9 Amplified linear fragment PCR reaction conditions.

Table S10 Overlap extension PCR reaction system of Δ*spoIIIJ*、Δ*spo0J*、Δ*kinA*.

Table S11 Overlap extension PCR reaction conditions Δ*spoIIIJ*、Δ*spo0J*、Δ*kinA*.

Table S12 Verification PCR reaction system Δ*spoIIIJ*、Δ*spo0J*、Δ*kinA*.

Table S13 Verification PCR reaction conditions Δ*spoIIIJ*、Δ*spo0J*、Δ*kinA*.

.

Table S1 The plasmids.

Strain	Genotype	Description	Reference/Source
Plasmids			
	pMD-19	Commercial T-Vector (Amp^R)	Takara, Maebashi, Japan
	pMarA	pUC19 carrying TnYLB-1 transposon mariner Himar1 transposase and promoter σA, spe^R	Lab store
	pCP16	pMD-19-*yuxH*-spe^R	This work
	pHB01	*spoIIIJ* complementary plasmid	This work
	pHB02	*spo0J* complementary plasmid	This work
	pHB03	*kinA* complementary plasmid	This work

Table S2 The Primers required for the amplification and validation of Δ*yuxH*、Δ*spoIIIJ*、Δ*spo0J*、Δ*kinA*.

Primers	（5’ - 3’）	amplification position	Strains
SLO1	AGGCAGAAATTCATCCCTTATTACTTTAAC	Upstream of *spoIIIJ*	Δ*spoIIIJ*
SLO2	GTACATCCGCAACTGTCCATCGCTGGCGGATGTTTCCCAATCC		
SLO3	CATAGTATCGACGGAGCCGAAAATCGTAACGATGATGATCGATAAAC	Downstream of *spoIIIJ*	
SLO4	AAATAAGGTTTCTAAACTGGTTAAGGTATGTT		
SLO13	AGTTAGGATTTATCCTCTTCTCTACAGAAA	Upstream of *spo0J*	Δ*spo0J*
SLO14	GTACATCCGCAACTGTCCATAAATAAAGACAAGCTTGAGCCGCTTG		
SLO15	CATAGTATCGACGGAGCCGATTTAGCGAGCTGTTCCTGGGTTAAATCCAA	Downstream of *spo0J*	
SLO16	ATGTGAAGGTAGGTGACATCGTGGGAA		
SLO21	GATCGGACAGTTTGATACGTATACGCC	Upstream of *kinA*	Δ*kinA*
SLO22	CATAGTATCGACGGAGCCGAATTACCTCGTCTTTGCTGTCGGC		
SLO23	GTACATCCGCAACTGTCCATATCACGACATTGTGAAAAACCGAATT	Downstream of *kinA*	
SLO24	CATGCAGACTATTTAAACAACGGCATAA		
spoIIIJ-F	TTCCTCCCGCTTTCTGCGTT	Validation of Δ*spoIIIJ*	Δ*spoIIIJ*

spoIIIJ-R	AAGATTGGTATAGGAGGAA		
spo0J-F	TTACGCTTCTCTTTCAGAC	Validation of Δ*spo0J*	Δ*spo0J*
spo0J-R	AAAGGCGGGCTCGGAAAAG		
kinA-F	GTGGAACAGGATACTCAG	Validation of Δ*kinA*	Δ*kinA*
kinA-R	GGTTGCAAGCATTAAAAGCTC		

Table S3 The reagents and enzymes.

The reagents and enzymes	Resource
TB Green® Premix Ex Taq™ II（Tli RNase H Plus）	RR820A，Takara
Prime Script™ RT Master Mix（Perfect Real Time）	RR036Q，Takara
Recombinant RNase Inhibitor	2313A，Takara
Recombinant DNase I（RNase-free）	2270A，Takara
Prime STAR® Max DNA Polymerase	R045Q, Takara
Rapid plasmid miniaturisation kit	DP105, Tian gen
Fast Pure Gel DNA Extraction Mini Kit	DC301-01, Vazyme
Premix Taq™ (Takara Taq™ Version 2.0 plus dye)	RR901A，Takara
Ready-to-use Seamless Cloning Kit	B632219-0020，Sheng gong

Table S4. Preparation of PCR reaction solution of Δ*yuxH*.

components	Volumes (μL)
Primer sense	1
Primer antisense	1
dNTP Mixture	2.5
10×PCR Buffer	2.5
Pfu DNA Polymerase	0.25
ddH_2O	up to 25

Table S5 PCR reaction Conditions of Δ*yuxH*.

procedure	T（℃）	time	cycle
stage1	95	30 s	1
	95	5 s	
stage2	60	30 s	30
	72	2 min	
	72	15 s	
stage3	10	5 min	1

Table S6 A-tailed reaction system for PCR products.

components	Volumes（μL）
PCR purified product	30
dNTP Mixture	5
10×PCR Buffer	5
Taq DNA Polymerase	0.5
ddH_2O	up to 50

Note: The reaction was carried out at 72 °C for 40 min.

Table S7 PCR product and linearized T-Vector pMD-19 ligation reaction system.

components	Volumes（μL）
Linearised carriers	1
Purpose fragment	1（0.1-0.3 pmol）
T4 DNA ligase	1
10×T4 DNA Buffer	1
ddH_2O	up to 10

Note: The ligation reaction was carried out at 16 °C for 2 h.

Table S8 Amplified linear fragment PCR reaction system.

components	Volumes（μL）
Template	2
Primer-F	2
Primer-R	2
PrimeSTAR® Max DNA Polymerase	25
ddH_2O	up to 50

Table S9 Amplified linear fragment PCR reaction conditions.

procedure	T（℃）	time	cycle
Pre-denaturation	98	10 sec	1
Denaturation	98	10 sec	30
Annealing	55	15 sec	30
Extension	72	5 sec/kb	30
Final extension	72	5 min	1

Table S10 Overlap extension PCR reaction system of Δ*spoIIIJ*、Δ*spo0J*、Δ*kinA*.

components	Volumes（μL）
Linear fragment Km	a
Linear fragment upstream	b
Linear fragment downstream	c
PrimeSTAR® Max DNA Polymerase	25

Note: Molar concentration a:b:c=1:1:1 and volume a+b+c=25 μL.

Table S11 Overlap extension PCR reaction conditions Δ*spoIIIJ*、Δ*spo0J*、Δ*kinA*.

procedure	T（℃）	time	cycle
Pre-denaturation	95	4 min	1
Denaturation	95	30 sec	12
Annealing	60	20 sec	12
Extension	72	5 sec/kb	12
Final extension	72	5 min	1

Table S12 Verification PCR reaction system Δ*spoIIIJ*、Δ*spo0J*、Δ*kinA*.

components	Volumes（μL）
Genome DNA	2
Primer-F	1
Primer-R	1
Premix Taq™(TaKaRa Taq™ Version2.0 plus dye)	12.5
ddH_2O	up to 25

Table S13 Verification PCR reaction conditions Δ*spoIIIJ*、Δ*spo0J*、Δ*kinA*.

procedure	T（℃）	time	cycle
Pre-denaturation	94	5 min	1
Denaturation	94	30 sec	30
Annealing	55	30 sec	30
Extension	72	2 min	30
Final extension	72	5 min	1
